# Different Apathy Profile in Behavioral Variant of Frontotemporal Dementia and Alzheimer's Disease: A Preliminary Investigation

**DOI:** 10.1155/2012/719250

**Published:** 2012-04-09

**Authors:** Davide Quaranta, Camillo Marra, Concettina Rossi, Guido Gainotti, Carlo Masullo

**Affiliations:** ^1^Istituto di Neurologia, Università Cattolica del Sacro Cuore, 00168 Rome, Italy; ^2^Neurology Unit, S. Giovanni Calibita Hospital (FBF), 00186 Rome, Italy

## Abstract

Apathy is one of the most common behavioral symptoms of dementia; it is one of the salient features of behavioral variant of frontotemporal dementia (bvFTD) but is also very frequent in Alzheimer's disease. This preliminary investigation was aimed at assessing the type of apathy-related symptoms in a population of bvFTD and AD subjects showing comparable apathy severity. Each patient underwent a comprehensive neuropsychological assessment; behavioral changes were investigated by the neuropsychiatric inventory (NPI), using the NPI-apathy subscale to detect apathetic symptoms. At univariate analysis, bvFTD subjects showed lack of initiation (*χ* ^2^ = 4.602, *p* = 0.032), reduced emotional output (*χ* ^2^ = 6.493, *p* = 0.008), and reduced interest toward friends and family members (*χ* ^2^ = 4.898, *p* = 0.027), more frequently than AD subjects. BvFTD displayed higher scores than AD on NPI total score (*p* = 0.005) and on subscales assessing agitation (*p* = 0.004), disinhibition (*p* = 0.007) and sleep disturbances (*p* = 0.025); conversely, AD subjects were more impaired on memory, constructional abilities, and attention. On multivariate logistic regression, reduced emotional output was highly predictive of bvFTD (OR = 18.266; *p* = 0.008). Our preliminary findings support the hypothesis that apathy is a complex phenomenon, whose clinical expression is conditioned by the site of anatomical damage. Furthermore, apathy profile may help in differentiating bvFTD from AD.

## 1. Introduction

Apathy has been repeatedly reported to be one of the most common noncognitive symptoms of dementia [1–3]. Frequency and severity of apathy vary across different dementia subtypes; it is the most common behavioral symptom of behavioral variant of frontotemporal dementia (bvFTD), with reported prevalence ranging from 62 to 89% of patients [4]; the prevalence of apathy in AD ranges from 25 to 88% [5, 6] with a trend to increase with disease severity [7]. When severity was directly compared, higher levels of apathy have been reported in bvFTD than in AD [8–11]. The functional and neuroanatomical substrates of apathy seem to differ between AD and bvFTD. In bvFTD, apathy severity has been associated with orbitofrontal abnormalities, both in MRI [12] and PET [13] studies, and with volume loss in the dorsal anterior cingulate and dorsolateral prefrontal cortex [14]. On the other hand, in AD apathy severity has been connected to neurofibrillary tangles density in the anterior cingulate gyrus [15] and to grey matter atrophy in the anterior cingulate and in the left medial frontal cortex [16]. These findings were confirmed by a PET study showing the association of apathy with hypometabolism in the bilateral anterior cingulate gyrus and medial orbitofrontal cortex [17].

On these grounds, it is quite clear that there is not a complete overlap between the anatomical substrates of apathy in bvFTD and AD, even though most of the previous studies have regarded apathy as an unitary complex. However, Marin [18–20] has proposed that apathy, defined as a “lack of motivation not attributable to diminished level of consciousness, cognitive impairment or emotional distress,” is a composite phenomenon, whose specific symptomatology can be dissected. “Affective-emotional” apathy would be characterized by a reduced ability to associate emotions to behaviors, manifesting as indifference or lack of empathy; “behavioral apathy” would be characterized by a reduction in spontaneous generation of motor patterns, so the patients need to be prompted to perform physical activities; finally, “cognitive apathy” would be characterized by an inactivation of goal-directed cognitive activity manifested by the need of external stimuli to start mental activity or speech [8, 18]. Levy and Dubois [21] proposed a different view of apathetic symptoms, stating that lack of motivation could be considered a projective and nonmeasurable construct, whereas apathy should be considered more correctly from a “behavioristic” point of view. They defined apathy as “the quantitative reduction of self-generated voluntary and purposeful behaviors” [21]. Accordingly, they proposed that apathy would be a pathology of voluntary action or goal-directed behavior, caused by dysfunctions occurring at the level of elaboration, execution, and control of goal-directed behavior [22]. The phenomenological distinction proposed by Levy and Dubois differs only slightly from the initial one proposed by Marin as they identified three dysfunctional domains: “affective-emotional,” “cognitive,” and “autoactivation”.

The distinction of “apathetic domains” may lead to the identification of specific neuroanatomical substrates for each of them. As a general observation, the occurrence of apathy is connected to damage of prefrontal cortex (PFC) and basal ganglia [5, 21]; thus, the segregation of the PFC-basal ganglia circuitry [23, 24] may represent the substrate of the different clinical phenotypes of apathy [21]: “emotional-affective” apathy may be related to the orbitomedial PFC and ventral striatum; “cognitive apathy” may be associated with dysfunction of lateral PFC and dorsal caudate nuclei; deficit of “autoactivation” may be due to bilateral lesions of the internal portion of globus pallidus, bilateral paramedian thalamic lesions, or the dorsomedial portion of PFC.

On these bases, it is conceivable that the apathetic symptoms shown by AD and bvFTD patients may be different from a qualitative point of view. This finding would also explain the different neuroanatomic substrates of apathy identified by structural and functional neuroimaging in bvFTD [12–14] and AD [15–17].

This hypothesis has been previously explored by Chow et al. [8]. These authors studied a large sample of AD and FTD subjects and reported that the clinical profile of apathy in FTD and AD was substantially overlapping. However, they observed that apathy was associated with different behavioral changes among the two groups of patients, namely compulsions and impulsivity in FTD, and dysphoria in AD.

The present preliminary investigation was aimed at assessing the apathy profile of bvFTD and AD and to assess its possible role in the differential clinical diagnosis, as compared to other behavioral changes and different neuropsychological patterns.

## 2. Materials and Methods

### 2.1. Subjects

**Figure 1:**
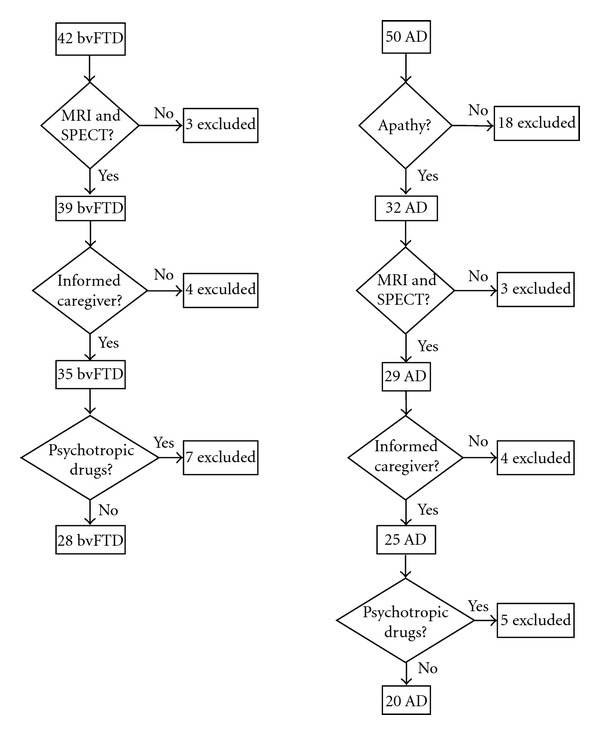
patients' selection work-flow. bvFTD: behavioral variant of frontotemporal dementia; AD: Alzheimer's disease.

Forty-two subjects fulfilling clinical diagnostic criteria for behavioral variant of frontotemporal dementia [25] were screened among subjects referring to our Neuropsychology Unit for memory and behavioral disorders. Exclusion criteria were, in addition to those provided by the corresponding diagnostic criteria, the absence of an informed caregiver, unavailability of neuroradiological examination, and/or the assumption of psychotropic drugs within two months prior to the clinical assessment. Following these exclusion criteria, four patients were excluded in consequence of lack of a sufficiently informed caregiver; three subjects were excluded because neuroimaging examinations were not available; finally, seven subjects were assuming psychotropic drugs (typical antipsychotics: 1 subject; atypical antipsychotics: 1 subject; antidepressants: 4 subjects; cholinesterase inhibitors: 1 subject) during the two months prior to our assessment (see Figure 1).

Additionally, twenty subjects affected by probable AD according to NINDCS-ADRDA criteria [26], matched to the bvFTD group for age and educational level, were selected. Furthermore, AD patients were matched to bvFTD patients even for apathy level in order to avoid an overestimation of symptoms frequency due to different levels of disease severity. Each patient underwent a complete medical and neurological examination. In order to be enrolled into the study subjects had to show on brain MRI the classical pattern of atrophy of bvFTD (frontal and temporal lobe atrophy) or AD (hippocampal atrophy) and display hypoperfusion in frontal or frontotemporal regions (bvFTD) or in temporo-parietal and precuneus regions (AD) on HMPAO-SPECT. The diagnosis was confirmed after 6 and 12 months of clinical follow-up.

### 2.2. Neuropsychological Assessment

Each patient underwent the Mini-Mental State Examination (MMSE) [27] and the Clinical Dementia Rating (CDR) scale [28]. Furthermore, patients were administered an extensive neuropsychological examination, including tasks of visual and verbal memory (Rey's Auditory Verbal Learning Test (RAVLT) including subtests of immediate and delayed recall and forced-choice recognition [29], Rey-Osterrieth Complex Figure (ROCF) recall [30]); phonological (F, A, S) and semantic (birds, furniture) verbal fluency (resp., PVF and SVF); confrontation naming of pictures of objects and actions; copy of Rey's complex figure [30], executive functions (Stroop's test [31], Frontal Assessment Battery (FAB) [32]); visual attention (Multiple Features Targets Cancellation (MFTC) [33]); abstract reasoning (Raven's Coloured Progressive Matrices—PM'47 [29]); copy of pictures with and without landmarks [29, 34].

### 2.3. Behavioral Assessment

Behavioral features were assessed by means of the Neuropsychiatric Inventory (NPI) [35], a well-known informant-based 12-domains questionnaire requiring the interview of the patient's primary caregiver (usually the spouse). The interview assessed the presence, frequency, and severity of twelve behavioral symptoms (viz., delusions, hallucinations, agitation, depression, anxiety, elation/euphoria, apathy, disinhibition, irritability, abnormal motor behavior, sleep disturbances, and appetite disturbances) commonly observed in demented patients. For each domain, the interview started with a screening question aimed at assessing the presence of abnormality in a specific behavior. If the caregiver reported an abnormal behavior, this was further explored with more specific questions. Frequency and severity were assessed separately. The total score ranged from 0 (no abnormalities) to 12 (severe abnormalities).

The apathy investigation was conducted by means of the NPI-apathy subscale which has been showed to be psychometrically robust across the range of different types of dementia [36]. Information was gathered about the presence of each subitem taken into account; the apathetic symptoms were coded on a presence/absence basis. The choice of the NPI-apathy subscale to assess apathetic symptoms was made for three principal reasons. First of all, the NPI is a widely used and well-known diagnostic tool, thus we were quite convinced that the possible observation of clear differences in apathy-related symptoms obtained by its administration could be easily applied and replicated in clinical practice; secondly, since the behavioral profile of bvFTD is complex and includes several typologies of disturbances, we reputed that it would be useful to assess apathy and other behavioral symptoms in a homogeneous way. Finally, the NPI-apathy subscale, together with the Apathy Evaluation Scale, has been reported to be the most robust assessment scale for apathy in patients with dementia [36].

### 2.4. Statistics

The small sample size and the use of discrete variables (NPI scores) have led to the use of nonparametric statistics; thus, comparison of continuous variable has been performed using the Mann-Whitney *U*-test, whereas *χ* ^2^-test with Yates' continuity correction was used to compare frequencies.

In order to verify the reliability of findings obtained in univariate statistics, a backward stepwise logistic regression analysis was performed, setting diagnosis (bvFTD versus AD) as the dependent variable and variables with significance level <0.05 at univariate analyses as predictors. The reliability of the regression analysis was assessed using the method described by Hosmer and Lemeshow [37]. Sensitivity, specificity, negative predictive value, positive predictive value, and area under the ROC curve (AROC) were determined for the logistic regression model in order to assess its diagnostic reliability. The significance level was two-sided for each statistical comparison.

## 3. Results

### 3.1. Sample Characteristics

Both bvFTD and AD patient groups were equal in age (resp., 66.25 ± 8.737 years versus 69.30 ± 7.828 years; |*z*| = 1.173, *p* = 0.241), education (11.29 ± 4.799 years versus 9.30 ± 4.181 years; |*z*| = 1.491, *p* = 0.136), and clinical duration of the disease (51.68 ± 34.020 months versus 36.65 ± 19.773 months; |*z*| = 1.600, *p* = 0.110). Furthermore, there were no differences in MMSE and CDR mean scores (Table 1).

### 3.2. Neuropsychological Assessment

Table 1 displays results of the neuropsychological evaluation. As expected, bvFTD performed better than AD patients on an episodic memory test (RAVLT delayed recall; *p* = 0.010) and on a test of constructional praxis (figure copy, *p* = 0.042). Furthermore, AD patient were slower than bvFTD patients on MFTC (*p* = 0.033), whereas bvFTD patients performed worse than AD patients on object naming (*p* = 0.029). Trendwise significance was observed for SVF (*p* = 0.065) and ROCF copy (*p* = 0.084).

### 3.3. Behavioral Examination

As easily predictable, patients affected by bvFTD showed more pronounced behavioral disturbances than AD (Table 2). In particular, bvFTD sample obtained higher NPI total score (*p* = 0.005) and higher scores on the subscales assessing agitation (*p* = 0.004), disinhibition (*p* = 0.007), and sleep disturbances (0.028). Statistical trends were detected also for euphoria (*p* = 0.056), abnormal motor behavior (*p* = 0.090), and appetite disturbances (*p* = 0.092).

### 3.4. Apathetic Symptoms

Table 3 reports the frequency of occurrence of the individual apathetic symptoms assessed by the NPI apathy subscale. bvFTD patients showed more frequently a reduction of conversation initiation (question no. 2, *p* = 0.032), behaved less affectionately and displayed lower emotional output (question no. 3, *p* = 0.008), and showed lower interest toward friends and family members (question no. 6, *p* = 0.027) than AD subjects. On the other hand, reduction of activity (question no. 1), lower participation in household chores (question no. 4), lost of interest in the activities of other persons (question no. 5) and in his/her own hobbies (question no. 7), and reduced care about new things (question no. 8) were reported with similar frequencies among the groups.

### 3.5. Multivariate Logistic Analysis

The logistic regression model included at the beginning block neuropsychological scores (RAVLT delayed recall, figure copy, MFTC time of execution, and objects naming), behavioral data (NPI: agitation, disinhibition, sleep disturbances, and NPI total score), and presence of apathetic symptoms (“yes” responses to NPI apathy subscale questions no. 2, 3, and 6). The dependent variable was the diagnosis, and odds ratios (OR) were calculated for the risk of bvFTD.

The final model included MFTC time of execution (OR = 0.975; 95%CI = 0.955−0.995; *p* = 0.016), “yes” response to question no. 3 (OR = 18.266; 95%CI = 2.531–131.792; *p* = 0.008) of the NPI-apathy subscale, and the score obtained on the objects naming (OR = 0.703; 95%CI = 0.551–0.896; *p* = 0.004). Following Hosmer and Lemeshow's method, the model goodness-of-fit was satisfactory (*χ* ^2^ = 28.34; *p* = 0.947).

The diagnostic accuracy of the model was good; 81.25% of the subjects were correctly classified, with sensitivity of 89.3%, specificity of 70.0%, PPV of 80.7%, and NPV of 82.3%; the AROC was 0.910.

## 4. Discussion

Apathy is a complex phenomenon, whose clinical architecture has been extensively investigated in recent years [18–22]. It also appears as the most common behavioral symptom of dementia [1–3], especially in bvFTD [4]. Furthermore, previous studies reported that subjects affected by bvFTD generally display higher levels of apathy as compared to AD patients [8–11], without substantial differences from the phenomenological point of view [8].

The main finding of the present study is the observation of a different distribution of apathetic symptoms between bvFTD and AD subjects matched for disease severity (as assessed by MMSE and CDR) and for severity of the apathetic symptomatology (as assessed by means of the total NPI-apathy score). In our samples, subjects affected by bvFTD displayed higher frequency of “affective” symptoms (NPI-apathy questions: “is the patient less affectionate or lacking in emotions when compared to his/her usual self?”; “has the patient lost interest in friends and family members?”), and a reduction of “auto-activation” [21] (or “behavioral apathy,” [18]) in comparison with AD sample.

The different clinical expression of apathy among the two groups of patients probably reflects the involvement of different anatomic substrates. Previous studies have reported that in bvFTD apathy is associated with changes in orbitofrontal cortex [12, 13], which, in turn, has been postulated to be the anatomical correlate of “affective” apathy [21]. Thus, it is possible that our observation may reflect an alteration of orbitofrontal cortex and its connections with subcortical nuclei (ventral striatum) that could be specific of bvFTD. We did not find such a dissociation as for the other apathetic symptoms taken into account and this may reflect the partial overlap of functional alterations between AD and bvFTD, particularly in the anterior cingulate gyrus [14, 17].

“Affective apathy” may be also regarded as the clinical expression of personality changes in bvFTD; for example, Sollberger et al. [38] reported that subjects with FTD and semantic dementia displayed a reduction in affiliative behavior (lack of warmth) and showed, in a large sample of subjects affected by different neurodegenerative diseases, an association between “warmth” and several cortical and subcortical right hemisphere structures (viz. orbitofrontal cortex, insular cortex, amygdala, and hippocampal and parahippocampal regions). This finding is of particular interest, since the authors reported an association between lack of warmth and cerebral structures related to reward mechanisms, and “affective apathy” has been regarded as consequence of the inability to associate emotions to behaviors [18–20]. Analogously, affective apathy may be related to an impairment of the so-called prosocial sentiments (such as guilt, pity, and embarrassment), connected to lack of empathy; Moll et al. [39] reported reduced social sentiments in bvFTD subjects; this deficit was related to hypometabolism in medial frontal polar cortex and septal area.

The results of our study support the hypothesis that apathy is a complex syndrome, with different clinical expressions across different pathological conditions. On the basis of our findings, it is conceivable that differences in qualitative aspects of apathy (and not in its severity) could be associated with differences in the damage site, as previously reported [21]. However, given the small size of our sample and the slight (yet not statistically significant) difference in overall apathy severity, we are not able to rule out that the site of damage may affect also the severity of apathy.

Another interesting finding of the present study is that a more detailed assessment of apathy, which is very common in bvFTD and AD, could contribute in differentiating these conditions. In fact, the presence of “affective” apathy was the only behavioral change able to distinguish bvFTD from AD patients in the multivariate regression model. It must be considered that we selected AD patients with apathy level comparable to bvFTD. This methodological approach was chosen because apathy is common in AD and is possibly present in the early phase of the disease [5, 6], even in MCI [40]. Therefore, we decided to explore the clinical scenario of subjects showing significant apathetic symptoms in association with cognitive changes that may be considered a relevant differential diagnostic challenge.

One could find surprising that neither memory disturbances nor executive functions were able to distinguish AD from bvFTD. However, we have previously reported that performances on typical executive tests may be similar between bvFTD and AD [10] and memory disturbances are common in bvFTD. Furthermore, the expected cognitive and behavioral differences were confirmed at univariate analysis. AD subjects resulted significantly in being more impaired in episodic memory and constructional abilities, whereas they displayed less behavioral disturbances. Thus, the association of affective apathy with bvFTD was strong enough to make most of the other differences lose their predictive role.

Our findings are at variance with the study conducted by Chow et al. [8] on the clinical features of apathy in FTD and AD. They reported that the phenomenology of apathy was similar between AD and FTD and that differences could be found only in its correlates with other behavioral disturbances. Nevertheless, in their study apathetic symptoms assessed by NPI were arbitrarily subdivided on the basis of Marin's model and a half of the domains (4 out of 8) were not unequivocally classified after expert consensus. Moreover, the perspective of the present paper has been quite different from the one of Chow et al. since they reported that AD subjects obtained lower scores on the NPI-apathy subscale than FTD patients, whereas our study was carried out to investigate differences in apathy profile when apathy severity was comparable. Finally, the FTD group enrolled by Chow et al. included 39 (42% of the FTD group) subjects affected by Primary Progressive Aphasia who are characterized by less severe and specific behavioral disturbances [10]. Therefore, the results of this study are hardly comparable with those previously reported by Chow et al. [8]. 

Obviously, our findings require confirmation from independent studies on larger series of subjects because the relatively small sample of AD and FTD patients taken into account in the present investigation can be considered as the main weakness of our research; furthermore, only a single question of the NPI-apathy subscale entered the final logistic regression model, alongside with the MFTC and naming task scores, thus resizing the predictive value of apathy-related symptoms in differential diagnosis between bvFTD and AD. However, it is worth noting that the overall diagnostic accuracy of the logistic regression model obtained in the present investigation (81.25%) is not far from results of previous studies that reported a diagnostic accuracy of about 85% on autopsy confirmed series, using complex neuropsychological battery [41].

The absence of data about the single-item reliability of NPI-apathy subscale could be regarded as another limitation of our study, leading to a cautious interpretation of the results. Nevertheless, we are quite confident that results of the present study support the view of apathy as a complex phenomenon, encompassing several clinical expressions, whose appearance is mainly related to the anatomical locus of damage. Furthermore, a refined investigation of apathy features could be useful in distinguishing bvFTD from AD when a relevant apathetic symptomatology is present.

## Figures and Tables

**Table 1:** Comparison of neuropsychological performances of the two groups. Statistically significant differences are indicated in bold; confounders included in the logistic regression analysis are reported in italics. MMSE: Mini-Mental Sate examination; CDR: Clinical Dementia Rating Scale; RAVLT: Rey's Auditory Verbal Learning Test; MFTC: Multiple Features Targets Cancellation; ROCF: Rey-Osterrieth Complex Figure.

	bvFTD (*N* = 28)		AD (*N* = 20)			
	Mean	SD	Mean	SD	|*z*|	*p*
MMSE	19.07	8.789	17.85	4.368	1.445	0.148
CDR	1.78	0.815	1.48	0.659	1.183	0.237
RAVLT immediate recall	20.59	12.858	17.45	7.702	1.005	0.315
RAVLT delayed recall	3.59	3.456	1.05	1.468	2.567	**0.010**
RAVLT recognition accuracy	77.37	22.047	72.00	16.403	1.616	0.106
RAVLT false alarms	8.89	8.916	11.10	7.137	1.352	0.176
Phonological verbal fluency	15.64	15.887	14.00	10.141	0.367	0.714
Semantic verbal fluency	6.79	6.373	9.00	4.634	1.849	*0.065*
Raven's colored matrices	18.18	7.822	15.94	7.630	1.340	0.180
Cube copy	2.65	1.285	1.90	1.242	2.034	**0.042**
Cube copy with landmarks	17.26	5.708	15.50	7.416	0.524	0.600
MFTC accuracy	80.71	24.424	73.33	19.538	1.401	0.161
MFTC time of execution	125.79	72.971	170.03	57.792	2.130	**0.033**
MFTC false alarms	5.96	9.796	9.65	12.874	1.174	0.241
Frontal assessment battery	7.88	6.124	8.30	2.726	0.199	0.842
Nouns denomination	17.85	8.198	23.01	4.382	2.180	**0.029**
Verbs denomination	13.52	7.223	16.95	5.562	1.492	0.136
ROCF copy	21.84	13.433	16.15	9.887	1.726	*0.084*
ROCF delayed reproduction	6.25	5.958	4.44	3.808	1.067	0.286
Stroop: interference/time	65.37	52.970	84.76	43.329	1.290	0.197
Stroop: interference/errors	10.72	11.353	15.11	10.907	1.418	0.156

**Table 2:** Comparison of behavioral profiles of the two groups. Statistically significant differences are indicated in bold; confounders included in the logistic regression analysis are reported in italics. NPI: Neuropsychiatric Inventory.

	bvFTD (*N* = 28)		AD (*N* = 20)			
	Mean	SD	Mean	SD	|*z*|	*p*
NPI delusions	1.89	3.72	1.05	1.701	0.324	0.746
NPI hallucinations	0.18	0.55	0.45	1.395	0.473	0.636
NPI agitation	3.48	4.07	1.00	2.026	2.897	**0.004**
NPI depression	2.54	3.05	1.80	1.542	0.245	0.806
NPI anxiety	2.93	3.79	1.95	3.300	0.873	0.382
NPI euphoria	1.79	3.11	0.60	2.088	1.912	*0.056*
NPI apathy	5.79	3.48	4.30	2.774	1.458	0.145
NPI disinhibition	2.07	3.13	0.45	1.395	2.697	**0.007**
NPI irritability	4.00	4.07	1.95	2.417	1.792	*0.073*
NPI aberrant motor behavior	3.29	4.23	1.20	2.215	1.697	*0.090*
NPI sleep disturbances	2.58	2.56	0.90	1.619	2.239	**0.025**
NPI appetite disturbances	4.79	4.53	2.60	3.393	1.687	0.092

NPI total score	3500	22.09	18.25	12.152	2.814	**0.005**

**Table 3:** Phenomenological features of apathy among FTD and AD subjects. Statistically significant differences are indicated in bold.

	bvFTD (*N* = 28)		AD (*N* = 20)			
	*N*	%	*N*	%	*χ* ^2^	*p*
(1) Does the patient seem less spontaneous and less active than usual?	4	14.3	4	20.0	0.017	0.896
(2) Is the patient less likely to initiate a conversation?	21	75.0	8	40.0	4.602	**0.032**
(3) Is the patient less affectionate or lacking in emotions when compared to his/her usual self?	19	67.9	5	25.0	6.943	**0.008**
(4) Does the patient contribute less to household chores?	17	60.7	11	55.0	0.010	0.921
(5) Does the patient seem less interested in the activities and plans of others?	19	67.9	12	60.0	0.065	0.799
(6) Has the patient lost interest in friends and family members?	20	71.4	7	35.0	4.898	**0.027**
(7) Is the patient less enthusiastic about his/her usual interests?	20	71.4	11	55.0	0.752	0.241
(8) Does the patient show any other signs that he/she does not care about doing new things?	15	53.6	6	30.0	1.763	0.184
